# Enhancing head and neck cancer detection accuracy in digitized whole-slide histology with the HNSC-classifier: a deep learning approach

**DOI:** 10.3389/fmolb.2025.1652144

**Published:** 2025-08-01

**Authors:** Haiyang Yu, Wang Yu, Yuan Enwu, Jun Ma, Xin Zhao, Linlin Zhang, Fang Yang

**Affiliations:** ^1^ Department of Laboratory Medicine, The Third Affiliated Hospital of Zhengzhou Univer-sity, Zhengzhou, Henan, China; ^2^ Zhengzhou Key Laboratory for In Vitro Diagnosis of Hypertensive Disorders of Pregnancy, Zhengzhou, China; ^3^ Tianjian Laboratory of Advanced Biomedical Sciences, Institute of Advanced Biomedical Sciences, Zhengzhou University, Zhengzhou, Henan, China; ^4^ The Radiology Department, The Third Affiliated Hospital of Zhengzhou University, Zhengzhou, Henan, China

**Keywords:** HNSCC, histopathology image, deep learning, cancer diagnosis, WSI

## Abstract

Head and neck squamous cell carcinoma (HNSCC) represents the sixth most common cancer worldwide, with pathologists routinely analyzing histological slides to diagnose cancer by evaluating cellular heterogeneity, a process that remains time-consuming and labor-intensive. Although no previous studies have systematically applied deep learning techniques to automate HNSCC TNM staging and overall stage prediction from digital histopathology slides, we developed an inception-ResNet34 convolutional neural network model (HNSC-Classifier) trained on 791 whole slide images (WSIs) from 500 HNSCC patients sourced from The Cancer Genome Atlas (TCGA) Head and Neck Squamous Cell dataset. Our pipeline was designed to distinguish cancerous from normal tissue and to predict both tumor stage and TNM classification from histological images, with the dataset split at the patient level to ensure independence between training and testing sets and performance evaluated using comprehensive metrics including receiver operating characteristic (ROC) analysis, precision, recall, F1-score, and confusion matrices. The HNSC-Classifier demonstrated exceptional performance with areas under the ROC curves (AUCs) of 0.998 for both cancer/normal classification and TNM system stage prediction at the tile level, while cross-validation showed high precision, recall, and F1 scores (>0.99) across all classification tasks. Patient-level classification achieved AUCs of 0.998 for tumor/normal discrimination and 0.992 for stage prediction, significantly outperforming existing approaches for cancer stage detection. Our deep learning approach provides pathologists with a powerful computational tool that can enhance diagnostic efficiency and accuracy in HNSCC detection and staging, with the HNSC-Classifier having potential to improve clinical workflow and patient outcomes through more timely and precise diagnoses, serving as an automated decision support system for histopathological analysis of HNSCC.

## 1 Introduction

Head and neck squamous cell carcinoma (HNSCC) is the sixth most common cancer worldwide (Johnson et al., 2020), accounting for more than 90% of head and neck malignancies (Mody et al., 2021). HNSCC originates from the squamous epithelium of the upper respiratory tract and digestive tract of the oral cavity, pharynx, and larynx, among which pharyngeal squamous cell carcinoma and laryngeal squamous cell carcinoma are most prevalent (Fang et al., 2022).

The prognosis and survival of HNSCC depend on many variables, including the presentation of the stage and the site of involvement (Cohen et al., 2018). The 5-year overall survival in patients with stage I–II disease is typically 70%–90% (Carvalho et al., 2005). Stage III-IV disease typically has a poorer prognosis; specific factors affecting treatment decisions include the primary site and stage, tumor histology, human papillomavirus status, and prognosis, as well as the patient’s performance status, comorbid health conditions, social and logistic factors, and preferences (Mody et al., 2021). Therefore, identification of the stage of HNSCC is extremely important for prognosis and treatment planning.

With the development of deep neural networks (DNNs), especially convolutional neural networks (CNNs), image recognition is increasingly used in medicine and cancer research. In the medical field, clinical images have been increasingly used to provide deep learning solutions for automated decision support systems for disease diagnosis, prognosis, personalized treatment, and other tasks that improve healthcare system efficiency (Chu et al., 2021). In oncology research, deep learning studies have shown initial success in classifying cell types and predicting treatment outcomes in cancer, including constructing prognostic signatures based on specific biomarkers for various cancer types (Wang et al., 2022). Predicting oral cancer recurrence (Exarchos et al., 2012), predicting oral cancer (Aubreville et al., 2017; Sharma and Om, 2015), detecting lymph node metastases from breast cancer (Ehteshami et al., 2017; Golden, 2017), classification of lung cancer types (Chen et al., 2021; Wang et al., 2020), evaluation of therapy response (Massaf et al., 2022; Wu et al., 2019), and cancer stage detection (Jakimovski and Davcev, 2019; Patil and Bellary, 2020).

Several studies have addressed cell typing and tissue classification in oral squamous cell carcinoma (OSCC) using WSIs (Folmsbee et al., 2018; Shaban et al., 2019; Das et al., 2020; Halicek et al., 2019; Gupta et al., 2019). Recent works have employed tumor-infiltrating lymphocyte (TIL) segmentation and classification in OSCC WSIs, and have successfully classified oral squamous cell carcinoma and the presence or absence of oral dysplasia. However, the analysis of digital whole slide images (WSIs) remains challenging due to extremely high resolution compared to other medical imaging modalities. Moreover, these studies do not focus on the prediction of clinical stage of cancer, which is important for determining the extent of cancer progression and for treatment planning.

In this study, we provide a transfer learning method to predict the clinical stage for HNSCC using deep learning. A recent literature review shows that this is the first work to detect the clinical stage for HNSCC based on WSIs. The major contributions of this paper are summarized as follows: (1) We implement classification methods for the task of classification in HNSCC and infer the stage of the tumor based on transfer learning with patient-level data splitting to ensure robust validation. (2) We present an easy-to-use Python package named HNSC-classifier, which provides an automated solution for detecting tumors and inferring cancer stages (https://github.com/yangfangs/HNSC-classifier).

## 2 Materials and methods

### 2.1 Datasets

A total of 791 frozen slides with hematoxylin and eosin (H&E) staining collected from 500 patients with head and neck squamous cell carcinoma were retrieved from the Cancer Genome Atlas (TCGA) (Hoadley et al., 2018). To ensure independence and representative distribution, we performed stratified patient-level splitting using combined variables: overall cancer stage (I-IV), primary tumor site (larynx, tongue, oral cavity, other), and TNM components. Using scikit-learn’s StratifiedShuffleSplit, we allocated 80% of patients (400 patients, 633 slides) to training and 20% (100 patients, 158 slides) to testing, maintaining similar distributions across all stratification variables (p > 0.05, chi-square test).

The 791 digital whole slide images (WSIs) were retrieved from the TCGA-HNSC project (Hoadley et al., 2018). The tissue slides contain 24 various sites of resection or biopsy (Supplementary Figure S1G). The training dataset that the hematoxylin and eosin (H&E) stained histopathology WSIs are mainly distributed in laryns (24.1%), tongue (25.8%), Overlapping lesion of lip, oral cavity and pharynx (16.5%). Depending on the resolution, the WSIs can be divided into different levels (from level 0 to level max) (Supplementary Figure S1A). In this study, we used the resolution of level 0 to extract the tiles for training. Because all WSIs can be extracted tiles for neural network training from the level 0 resolution. In the TCGA-HNSC project, the highest resolution level 3 includes a total of 202 WSIs, level 2 includes 695 WSIs, level 1 includes 775 WSIs and level 0 includes all WSIs (Supplementary Figure S1B). The pixel distribution of WSIs at different level resolutions is shown in Supplementary Figure S1C. The training dataset of hematoxylin and eosin (H&E) stained histopathology WSIs are mainly distributed in larynx (24.1%), tongue (25.8%), and overlapping lesions of lip, oral cavity and pharynx (16.5%) (Table 1). The dataset exhibits notable class imbalance, particularly in advanced disease stages (T4b: 0.5%, IVC: 1.3%). To address this limitation, we implemented several strategies: (1) class-weighted loss functions with weights inversely proportional to class frequency, (2) stratified sampling during training to ensure balanced representation, and (3) evaluation on balanced test subsets for unbiased performance assessment. Categories with fewer than 10 samples were combined with similar stages for more robust training (T4a and T4b were grouped as T4+, and IVC cases were analyzed separately as metastatic disease).

**TABLE 1:** Distribution of TNM staging and cancer stages in the dataset.

TNM component	Category	Count	Percentage
T-stage	T1	37	7.1%
	T2	148	28.6%
	T3	138	26.6%
	T4	25	4.8%
	T4a	154	29.8%
	T4b	3	0.5%
	TX	12	2.3%
N-stage	N0	242	46.8%
	N1	84	16.3%
	N2	19	3.6%
	N2a	17	3.2%
	N2b	84	16.3%
	N2c	44	8.5%
	N3	9	1.7%
	NX	17	3.2%
M-stage	M0	486	94.38%
	M1	21	4%
	MX	6	1.2%
Overall Stage	I	21	4.1%
	II	95	18.7%
	III	106	20.9%
	IVA	267	52.6%
	IVB	11	2.1%
	IVC	7	1.3%

All WSIs were available at level 0 resolution (×40 magnification, approximately 0.25 μm/pixel), which was used for tile extraction to ensure consistent high-resolution analysis across all samples.

### 2.2 Tile extraction and preprocessing

We extracted non-overlapping tiles of 224 × 224 pixels via grid sampling for each WSI at level 0 resolution (×40 magnification) (Figure 2A). Tile sampling was performed using tissue-foreground detection to exclude background regions. Each tile was labeled as normal or tumor based on pathologist annotations available in the TCGA dataset. Color normalization was applied using the Macenko method to standardize staining variations across different institutions and scanning protocols. Data augmentation during training included random rotations (±15°), horizontal and vertical flips, and color jittering to improve model robustness.

### 2.3 Deep learning architecture and training

The classification of HNSCC with slide-level labels was formulated as a multi-instance learning problem. We implemented an ResNet34 architecture using the fastai2 (Howard and Gugger, 2020) deep learning framework. The model was pre-trained on ImageNet and fine-tuned for histopathology classification using transfer learning.

Architecture Details: The slide-level classification head consisted of:

• Global average pooling of tile-level features
• Two fully connected layers (512 and 256 neurons) with ReLU activation
• Dropout layers (p = 0.5) for regularization
• Final classification layer with softmax activation

Training Configuration:

• Optimizer: Adam with learning rate = 1e-4, weight decay = 1e-4
• Batch size: 32 tiles per batch
• Training epochs: 20
• Hardware: Single NVIDIA RTX A4000 GPU (16 GB memory)
• Loss function: Cross-entropy loss with class weighting to address imbalance

For slide-level prediction, we aggregated tile-level predictions using majority voting, where a slide was classified based on the predominant prediction across its constituent tiles. Early stopping was implemented with patience = 5 epochs monitoring validation loss to prevent overfitting. Training was set for a maximum of 20 epochs, though most models converged between epochs 15–18. The best model weights were restored from the epoch with minimum validation loss.

### 2.4 Addressing class imbalance

Given the significant class imbalance (1,399,566 tumor tiles vs. 52,579 normal tiles), we implemented several strategies: Class-weighted loss function with weights inversely proportional to class frequency. Stratified sampling to ensure balanced representation during training. Evaluation on balanced test subsets to provide unbiased performance metrics.

### 2.5 Performance measures

To evaluate HNSC-classifier performance, we conducted 10-fold cross-validation on the training set for model selection and hyperparameter tuning. The final model was evaluated on the independent test set. We used comprehensive metrics including receiver operating characteristic (ROC) curves, area under the ROC curve (AUC), true positive rate (TPR, recall) (Equation 1), precision (positive predictive value, PPV) (Equation 2), false positive rate (FPR) (Equation 3), accuracy (ACC) (Equation 4), F1 score (Equation 5) calculate as follow:

$$Recall=TPR=\frac{TP}{TP+FN}$$ (1)

$$precision=PPV=\frac{TP}{TP+FP}$$ (2)

$$FPR=\frac{FP}{FP+TN}$$ (3)

$$ACC=\frac{TP+TN}{TP+TN+FP+FN}$$ (4)

$$F1=\frac{2TP}{2TP+FP+FN}$$ (5)

Both tile-level and patient-level performance metrics were calculated to provide comprehensive evaluation. Patient-level classification was determined by slide-level predictions aggregated across all slides per patient. All statistical analyses and figure plots were performed using R (version 4.2.1) with packages “ggplot2”, “ggpubr” (Statistical significance was set at p < 0.05.) and Python (version 3.10.7).

## 3 Results

### 3.1 The scheme of the HNSC-Classifier

**FIGURE 1:**
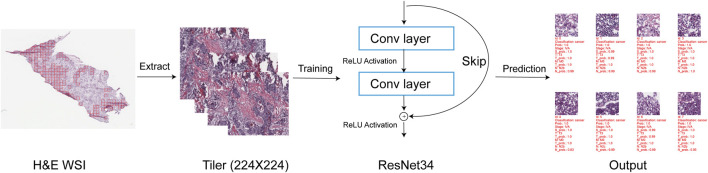
Schematic overview of the HNSC-Classifier pipeline. Workflow illustration showing: (1) H&E WSI tiling into 224 × 224 pixel patches, (2) ResNet34-based tile-level classification, (3) majority voting aggregation of tile predictions for slide-level classification, and (4) final patient-level diagnosis integration. The multi-instance learning approach aggregates individual tile predictions to generate robust slide-level cancer detection and staging predictions.

Figure 1 illustrates the workflow of the HNSC-classifier. The slide images were initially extracted into non-overlapping 224 × 224 tiles, which were used as instances for the subsequent training procedure. To determine the optimal method for training the ResNet34 model, we evaluated the performance of different pixel extraction methods and resolutions using a standard deep learning approach. ResNet34, a 34-layer residual learning framework, was selected as the model for HNSCC classification due to its proven effectiveness as a medical image classification model (He et al., 2016). The HNSC-classifier utilizes a pre-trained model to predict input HNSCC WSIs. The prediction output includes a summary of the prediction, the probability of prediction, and a detailed table with each tile’s prediction.

### 3.2 The performance of classification for tumor detection

**FIGURE 2:**
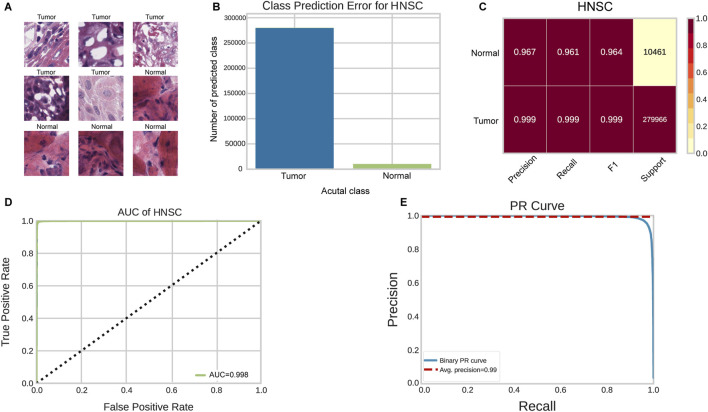
Performance evaluation of HNSC-Classifier for cancer detection. **(A)** Representative images of tumor and normal tissue tiles extracted from HNSCC WSIs. **(B)** Classification error analysis for tumor/normal prediction. **(C)** Performance metrics (precision, recall, F1 score) for tumor/normal tissue classification. **(D)** ROC curve analysis showing AUC of 0.998 for tumor/normal discrimination. **(E)** Precision-recall curve demonstrating classifier performance across different thresholds.

We utilized the ResNet34 image classification model to train the WSIs in this study. A total of 1,399,566 tumor tiles and 52,579 normal tiles were extracted from 791 WSIs as instances for the ResNet34 training (Figure 2A). To address the significant class imbalance, we applied class weighting and evaluated performance on balanced test subsets. The class prediction error showed minimal classification errors in the HNSCC tumor/normal classification test (Figure 2B). On balanced test data, the ResNet34 model achieved precision, recall, and F1 scores of 0.997, 0.997, and 0.997 for tumor detection, and 0.963, 0.965, and 0.963 for normal tissue classification, respectively (Figures 2C–E) (Supplementary Table S1). The ROC curve analysis showed an AUC of 0.998 for tile-level tumor/normal discrimination (Figure 2D). These results indicate that the deep-learning-based HNSC-classifier could be a useful tool for quantitative analysis of cancer tissue slides. We compared the ResNet34 model with other popular architectures including GoogLeNet (Szegedy et al., 2016), VGGNet (Qassim et al., 2018). The results showed that ResNet34 achieved the best balance between accuracy and computational efficiency (Supplementary Table S6).

### 3.3 The performance of classification for stage prediction

**FIGURE 3:**
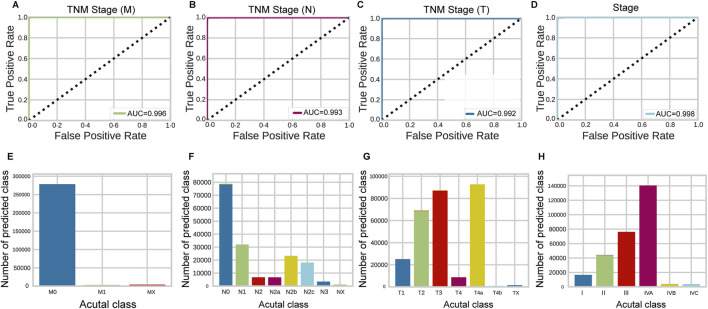
Stage prediction performance of HNSC-Classifier. **(A–D)** ROC curves with AUC values for predicting M, N, T components of the TNM system and overall cancer stage, respectively. **(E–H)** Classification error analysis for M, N, T, and stage system predictions, showing minimal prediction errors across all categories.

**FIGURE 4:**
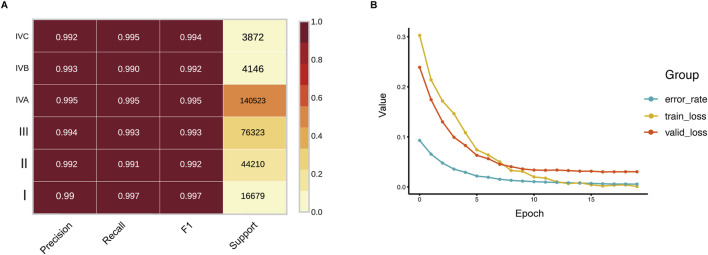
Training dynamics and performance metrics for stage prediction. **(A)** Precision, recall, and F1 scores for stage classification, demonstrating robust prediction across all cancer stages. **(B)** Training and validation loss curves along with error rate across 20 epochs, showing rapid convergence and minimal overfitting.

Figure 3 presents the performance of the HNSC-classifier in classifying different stages of cancer. Cancer diagnosis is usually determined by the stage at which it is diagnosed, and accurately predicting cancer staging based on WSIs can help pathologists determine cancer staging and facilitate treatment planning. The AUC for the ROC curves were 0.996, 0.993, and 0.992 for predicting M, N, and T stages, respectively (Figures 3A–C). The class prediction error demonstrated minimal misclassification in the HNSCC TNM staging system classification (Figures 3E–G). Patient-level TNM classification achieved AUCs of 0.994, 0.990, and 0.988 for M, N, and T stages, respectively. The AUC for the ROC curve was 0.998 at tile level and 0.992 at patient level for predicting different cancer stages (Figure 3D). The class prediction error indicated excellent performance across all stage categories (Figure 3H). The performance showed that precision, recall, and F1 scores were greater than 0.99 for each stage at tile level, and greater than 0.99 for each stage at patient level (Figure 4A) (Supplementary Table S2). The error rate and loss curves for the 34-layer residual learning model showed that the error rate decreased rapidly in the first 10 epochs and gradually reached convergence after 15 epochs (Figure 4B). Additional results of collected WSIs extracted tiles, precision, recall, and F1 score, error rate, loss of train and valid for the TNM system can be found in Supplementary Figure S2 (Supplementary Table S3), Supplementary Figure S3 (Supplementary Table S4), and Supplementary Figure S4 (Supplementary Table S5).

### 3.4 The effect of slide resolution and tile size on performance

**FIGURE 5:**
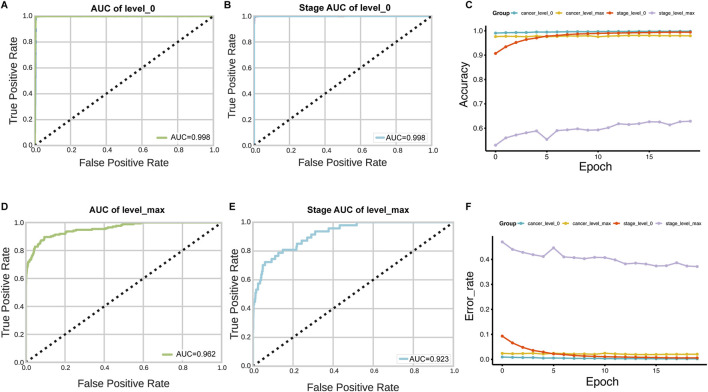
Impact of WSI resolution level on classification performance. **(A–D)** AUC comparison for tumor/normal classification using tiles extracted at level 0 (highest resolution) versus level max (lowest resolution). **(B–E)** Stage prediction AUC comparison between level 0 and level max. **(C–F)** Accuracy and error rate analysis across 20 training epochs for models trained with different resolution levels, demonstrating superior performance with level 0 data.

**FIGURE 6:**
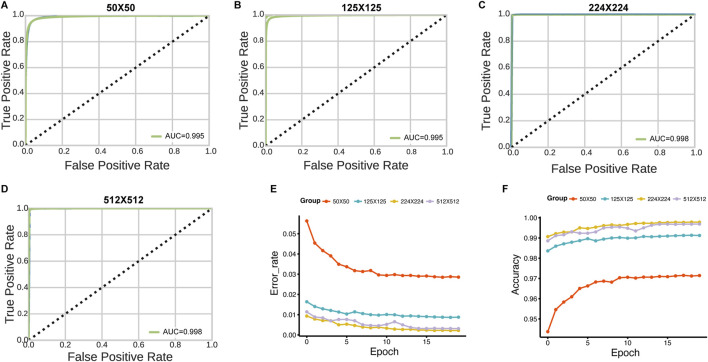
Effect of tile size on HNSC-Classifier performance. **(A–D)** ROC curves and AUC values for tumor/normal classification using tiles of varying sizes (50 × 50, 125 × 125, 224 × 224, and 512 × 512 pixels). **(E)** Error rate comparison across 20 training epochs for models trained with different tile sizes. **(F)** Accuracy comparison showing optimal performance with 224 × 224 pixel tiles.

The slides were available at different resolution levels. We tested the impact of slide resolution and tile size on classification accuracy. Level ×0(highest resolution, ×40 magnification) consistently outperformed lower resolution levels for both tumor/normal classification (AUC = 0.998 vs. 0.962 for level max) and stage prediction (AUC = 0.998 vs. 0.923 for level max) (Figure 5). For tile size optimization, we evaluated 50 × 50, 125 × 125, 224 × 224, and 512 × 512 pixel tiles. The optimal performance was achieved with 224 × 224 pixel tiles, which provided the best balance between detail preservation and computational efficiency (Figure 6).

## 4 Discussion

In this study, we developed a novel approach for cancer detection and stage diagnosis using a ResNet34 CNN architecture. Our deep learning-based classifier was utilized to differentiate the TNM system and stage system for HNSCC with patient-level data splitting to ensure robust validation. To the best of our knowledge, this is the first study to explore the HNSCC stage system in digitized WSIs utilizing the TCGA-HNSC project with comprehensive patient-level evaluation. The findings demonstrate that our method exhibits exceptional performance in cancer classification and stage inference. The HNSC-classifier developed in our study could aid clinicians in identifying cancer and determining the stage through WSIs, thus providing deeper insight into the prognostic and therapeutic importance of tumor subclass composition in HNSCC.

Histopathology is a widely used method for cancer diagnosis and is an essential part of medical protocols. However, examination of whole histopathological images can be a bottleneck in delivering timely treatment. For examination of whole slide images (WSIs) and determining cancer stage, pathologists often face heavy workloads, which can delay cancer patient treatment. In clinical practice, HNSCC detection, segmentation, and classification are carried out manually using WSIs stained with H&E under high-power microscopy, which is labor-intensive and time-consuming. Recent studies indicate that experienced pathologists require 10–20 min for comprehensive WSI review in digital pathology workflows (Wang et al., 2020), though this varies significantly based on case complexity and diagnostic features.

The scope of computational methods in medical applications continues to expand beyond image analysis. These approaches have demonstrated success across diverse medical domains, from pharmacophore-based virtual screening and molecular dynamics simulations for drug discovery to automated pathological image analysis (Du et al., 2020). The common strength of these computational techniques lies in their ability to process complex, high-dimensional datasets and provide objective, quantitative insights to support clinical decision-making.

Deep learning provides accurate classification, and for very large image datasets, it can exceed human classification levels (Dey et al., 2017). Therefore, computer-assisted diagnosis can be a fast and effective way to identify cancer and determine its stage. We present the HNSC-classifier, which implements a ResNet34 CNN architecture in Python, providing an easy-to-use and accurate HNSCC cancer classification and stage inference tool to alleviate expert burden.

The high performance metrics achieved in this study (AUC >0.99) are encouraging but require validation on independent, multi-institutional datasets to demonstrate generalizability across different scanners, staining protocols, and patient populations. The use of frozen sections, while providing high-quality morphological detail, may limit immediate clinical applicability, as routine diagnostic workflows typically use formalin-fixed paraffin-embedded (FFPE) specimens.

Future work should include validation on FFPE specimens, external validation cohorts, and prospective clinical studies to establish real-world effectiveness. Additionally, integration with existing pathology information systems and development of user-friendly interfaces will be crucial for clinical adoption.

Digital pathology using WSI allows pathologists to view high-resolution tissue images, and digital pathology is not inferior to microscopy for primary diagnosis (Mukhopadhyay et al., 2018; Pantanowitz et al., 2013). The current work shows potential for clinical benefit, as this study is based on deep learning training of frozen slides that provide excellent morphological preservation.

A significant limitation affecting immediate clinical applicability is our use of frozen sections, while routine diagnostic workflows predominantly use formalin-fixed paraffin-embedded (FFPE) specimens. FFPE processing introduces morphological changes and artifacts that may affect model performance. Additionally, our single-institution scanner data may not generalize across different imaging systems and staining protocols. Prospective multi-institutional validation studies using FFPE specimens from diverse clinical settings are essential before clinical implementation.

## Data Availability

The deep learning model created during this study is available at https://github.com/yangfangs/HNSC-classifier.

## Abbreviations

HNSCC, head and neck squamous cell carcinoma; WSIs, whole slide images; TCGA, the Cancer Genome Atlas; TCGA-HNSC, Head and neck squamous cell dataset; H&E, hematoxylin and eosin; ROC, receiver operating characteristic curve; TPR, true positive rate; FPR, false positive rate; AUC, area under the ROC curve; TP, true positive; FP, false positives; FN, false negatives; TN, true negatives; FFPE, Formalin-Fixed Paraffin-Embedded.

## References

[B1] Aubreville M. Knipfer C. Oetter N. Jaremenko C. Rodner E. Denzler J. (2017). Automatic classification of cancerous tissue in laserendomicroscopy images of the oral cavity using deep learning. Sci. Rep. 7 (1), 11979. 10.1038/s41598-017-12320-8 28931888 PMC5607286

[B2] Carvalho A. L. Nishimoto I. N. Califano J. A. Kowalski L. P. (2005). Trends in incidence and prognosis for head and neck cancer in the United States: a site-specific analysis of the SEER database. Int. J. Cancer 114 (5), 806–816. 10.1002/ijc.20740 15609302

[B3] Chen C. L. Chen C. C. Yu W. H. Chen S. H. Chang Y. C. Hsu T. I. (2021). An annotation-free whole-slide training approach to pathological classification of lung cancer types using deep learning. Nat. Commun. 12 (1), 1193. 10.1038/s41467-021-21467-y 33608558 PMC7896045

[B4] Chu C. S. Lee N. P. Ho J. W. Choi S.-W. Thomson P. J. (2021). Deep learning for clinical image analyses in oral squamous cell carcinoma: a review. JAMA Otolaryngology–Head & Neck Surg. 147 (10), 893–900. 10.1001/jamaoto.2021.2028 34410314

[B5] Cohen N. Fedewa S. Chen A. Y. (2018). Epidemiology and demographics of the head and neck cancer population. Oral Maxillofac. Surg. Clin. North Am. 30 (4), 381–395. 10.1016/j.coms.2018.06.001 30078696

[B6] Das N. Hussain E. Mahanta L. B. (2020). Automated classification of cells into multiple classes in epithelial tissue of oral squamous cell carcinoma using transfer learning and convolutional neural network. Neural Netw. 128, 47–60. 10.1016/j.neunet.2020.05.003 32416467

[B7] Dey D. Chatterjee B. Dalai S. Munshi S. Chakravorti S. (2017). A deep learning framework using convolution neural network for classification of impulse fault patterns in transformers with increased accuracy. IEEE Trans. Dielectr. Electr. Insulation 24 (6), 3894–3897. 10.1109/tdei.2017.006793

[B8] Du S. Yang B. Wang X. Li W.-Y. Lu X.-H. Zheng Z.-H. (2020). Identification of potential leukocyte antigen-related protein (PTP-LAR) inhibitors through 3D QSAR pharmacophore-based virtual screening and molecular dynamics simulation. J. Biomol. Struct. Dyn. 38 (14), 4232–4245. 10.1080/07391102.2019.1676825 31588870

[B9] Ehteshami B. B. Veta M. Johannes van Diest P. van Ginneken B. Karssemeijer N. Litjens G. (2017). Diagnostic assessment of deep learning algorithms for detection of lymph node metastases in women with breast cancer. JAMA 318 (22), 2199–2210. 10.1001/jama.2017.14585 29234806 PMC5820737

[B10] Exarchos K. P. Goletsis Y. Fotiadis D. I. (2012). Multiparametric decision support system for the prediction of oral cancer reoccurrence. IEEE Trans. Inf. Technol. Biomed. 16 (6), 1127–1134. 10.1109/TITB.2011.2165076 21859630

[B11] Fang Y. Yang Y. Liu C. (2022). Evolutionary relationships between dysregulated genes in oral squamous cell carcinoma and oral microbiota. Front. Cell. Infect. Microbiol. 987. 10.3389/fcimb.2022.931011 PMC9328420 35909962

[B12] Folmsbee J. Liu X. Brandwein-Weber M. Doyle S. (2018). “Active deep learning: improved training efficiency of convolutional neural networks for tissue classification in oral cavity cancer,” in 2018 IEEE 15th international symposium on biomedical imaging (ISBI 2018): 2018 (IEEE), 770–773.

[B13] Golden J. A. (2017). Deep learning algorithms for detection of lymph node metastases from breast cancer: helping artificial intelligence Be seen. JAMA 318 (22), 2184–2186. 10.1001/jama.2017.14580 29234791

[B14] Gupta R. K. Kaur M. Manhas J. (2019). Tissue level based deep learning framework for early detection of dysplasia in oral squamous epithelium. J. Multimedia Inf. Syst. 6 (2), 81–86. 10.33851/jmis.2019.6.2.81

[B15] Halicek M. Shahedi M. Little J. V. Chen A. Y. Myers L. L. Sumer B. D. (2019). Head and neck cancer detection in digitized whole-slide histology using convolutional neural networks. Sci. Rep. 9 (1), 14043–11. 10.1038/s41598-019-50313-x 31575946 PMC6773771

[B16] He K. Zhang X. Ren S. Sun J. (2016). “Deep residual learning for image recognition,” in Proceedings of the IEEE conference on computer vision and pattern recognition, 770–778.

[B17] Hoadley K. A. Yau C. Hinoue T. Wolf D. M. Lazar A. J. Drill E. (2018). Cell-of-Origin patterns dominate the molecular classification of 10,000 tumors from 33 types of cancer. Cell 173 (2), 291–304.e6. 10.1016/j.cell.2018.03.022 29625048 PMC5957518

[B18] Howard J. Gugger S. (2020). Fastai: a layered API for deep learning. Information 11 (2), 108. 10.3390/info11020108

[B19] Jakimovski G. Davcev D. (2019). Using double convolution neural network for lung cancer stage detection. Appl. Sci. 9 (3), 427. 10.3390/app9030427

[B20] Johnson D. E. Burtness B. Leemans C. R. Lui V. W. Y. Bauman J. E. Grandis J. R. (2020). Head and neck squamous cell carcinoma. Nat. Rev. Dis. Prim. 6 (1), 92. 10.1038/s41572-020-00224-3 33243986 PMC7944998

[B21] Massafra R. Comes M. C. Bove S. Didonna V. Gatta G. Giotta F. (2022). Robustness evaluation of a deep learning model on sagittal and axial breast DCE-MRIs to predict pathological complete response to neoadjuvant chemotherapy. J. Pers. Med. 12 (6), 953. 10.3390/jpm12060953 35743737 PMC9225219

[B22] Mody M. D. Rocco J. W. Yom S. S. Haddad R. I. Saba N. F. (2021). Head and neck cancer. Lancet 398 (10318), 2289–2299. 10.1016/S0140-6736(21)01550-6 34562395

[B23] Mukhopadhyay S. Feldman M. D. Abels E. Ashfaq R. Beltaifa S. Cacciabeve N. G. (2018). Whole slide imaging versus microscopy for primary diagnosis in surgical pathology: a multicenter blinded randomized noninferiority study of 1992 cases (pivotal study). Am. J. Surg. Pathol. 42 (1), 39–52. 10.1097/PAS.0000000000000948 28961557 PMC5737464

[B24] Pantanowitz L. Sinard J. H. Henricks W. H. Fatheree L. A. Carter A. B. Contis L. (2013). Validating whole slide imaging for diagnostic purposes in pathology: guideline from the College of American Pathologists Pathology and Laboratory Quality Center. Arch. Pathol. Lab. Med. 137 (12), 1710–1722. 10.5858/arpa.2013-0093-CP 23634907 PMC7240346

[B25] Patil R. Bellary S. (2020). Machine learning approach in melanoma cancer stage detection. Journal of King Saud University-Computer and Information Sciences. 34.6 (2022), 3285–3293.

[B26] Qassim H. Verma A. Feinzimer D. (2018). “Compressed residual-VGG16 CNN model for big data places image recognition,” in 2018 IEEE 8th annual computing and communication workshop and conference (CCWC) (IEEE), 169–175.

[B27] Shaban M. Khurram S. A. Fraz M. M. Alsubaie N. Masood I. Mushtaq S. (2019). A novel digital score for abundance of tumour infiltrating lymphocytes predicts disease free survival in oral squamous cell carcinoma. Sci. Rep. 9 (1), 13341. 10.1038/s41598-019-49710-z 31527658 PMC6746698

[B28] Sharma N. Om H. (2015). Usage of probabilistic and general regression neural network for early detection and prevention of oral cancer. ScientificWorldJournal 2015, 234191. 10.1155/2015/234191 26171415 PMC4485993

[B29] Szegedy C. Vanhoucke V. Ioffe S. Shlens J. Wojna Z. (2016). “Rethinking the inception architecture for computer vision,” in Proceedings of the IEEE conference on computer vision and pattern recognition, 2818–2826.

[B30] Wang L. Qiu M. Wu L. Li Z. Meng X. He L. (2022). Construction and validation of prognostic signature for hepatocellular carcinoma basing on hepatitis B virus related specific genes. Infect. Agents Cancer 17 (1), 60. 10.1186/s13027-022-00470-y PMC9727957 36474267

[B31] Wang X. Chen H. Gan C. Lin H. Dou Q. Tsougenis E. (2020). Weakly supervised deep learning for whole slide lung cancer image analysis. IEEE Trans. Cybern. 50 (9), 3950–3962. 10.1109/TCYB.2019.2935141 31484154

[B32] Wu E. Hadjiiski L. M. Samala R. K. Chan H. P. Cha K. H. Richter C. (2019). Deep learning approach for assessment of bladder cancer treatment response. Tomography 5 (1), 201–208. 10.18383/j.tom.2018.00036 30854458 PMC6403041

