# Supplementary figures and images for: Enhancing head and neck cancer detection accuracy in digitized whole-slide histology with the HNSC-classifier: a deep learning approach
Source: Front Mol Biosci. 2025 Aug 1;12:1652144. doi: 10.3389/fmolb.2025.1652144 (PMC12353728; doi:10.3389/fmolb.2025.1652144)

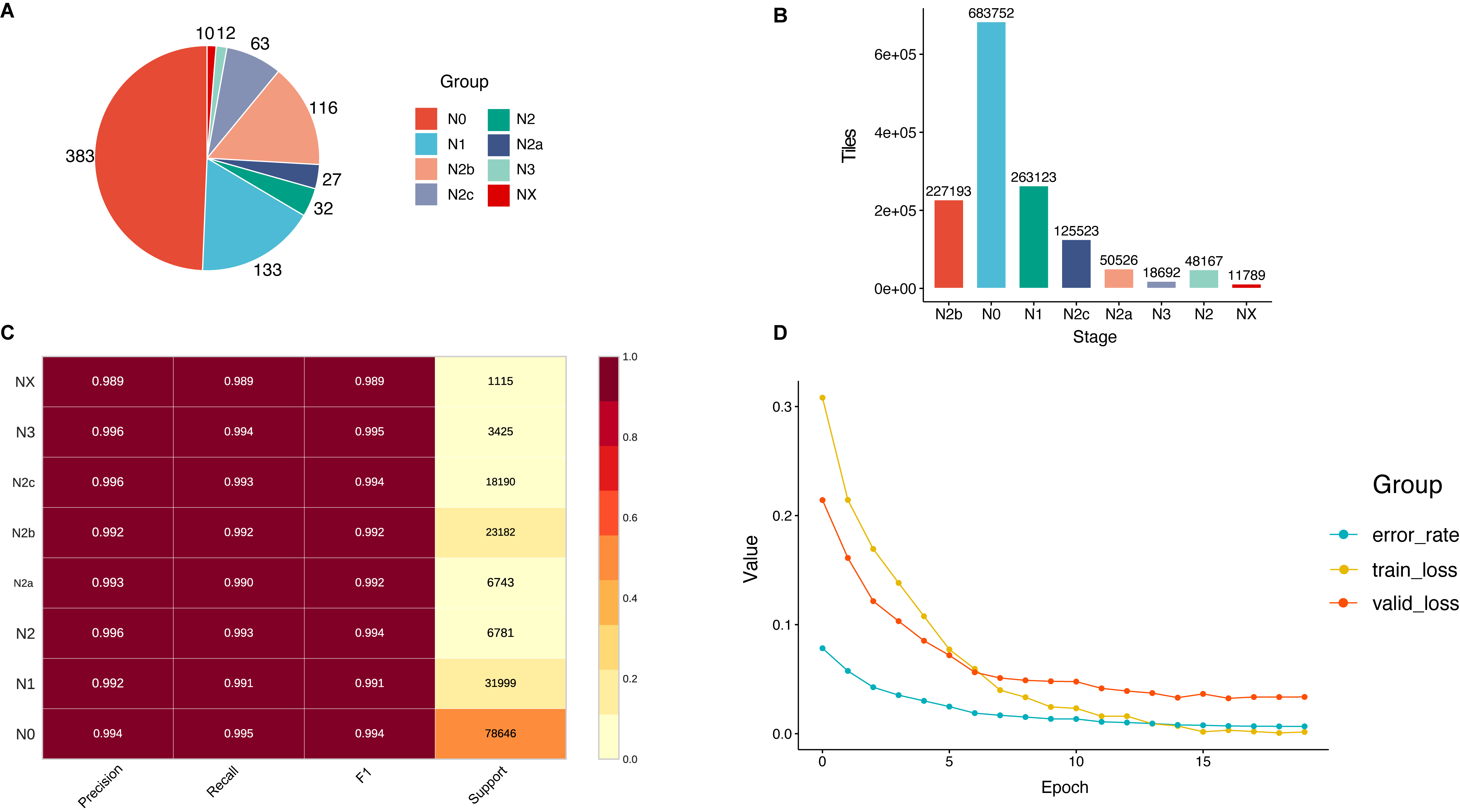

Supplement: Supplementary file 2 [file Image3.tif]

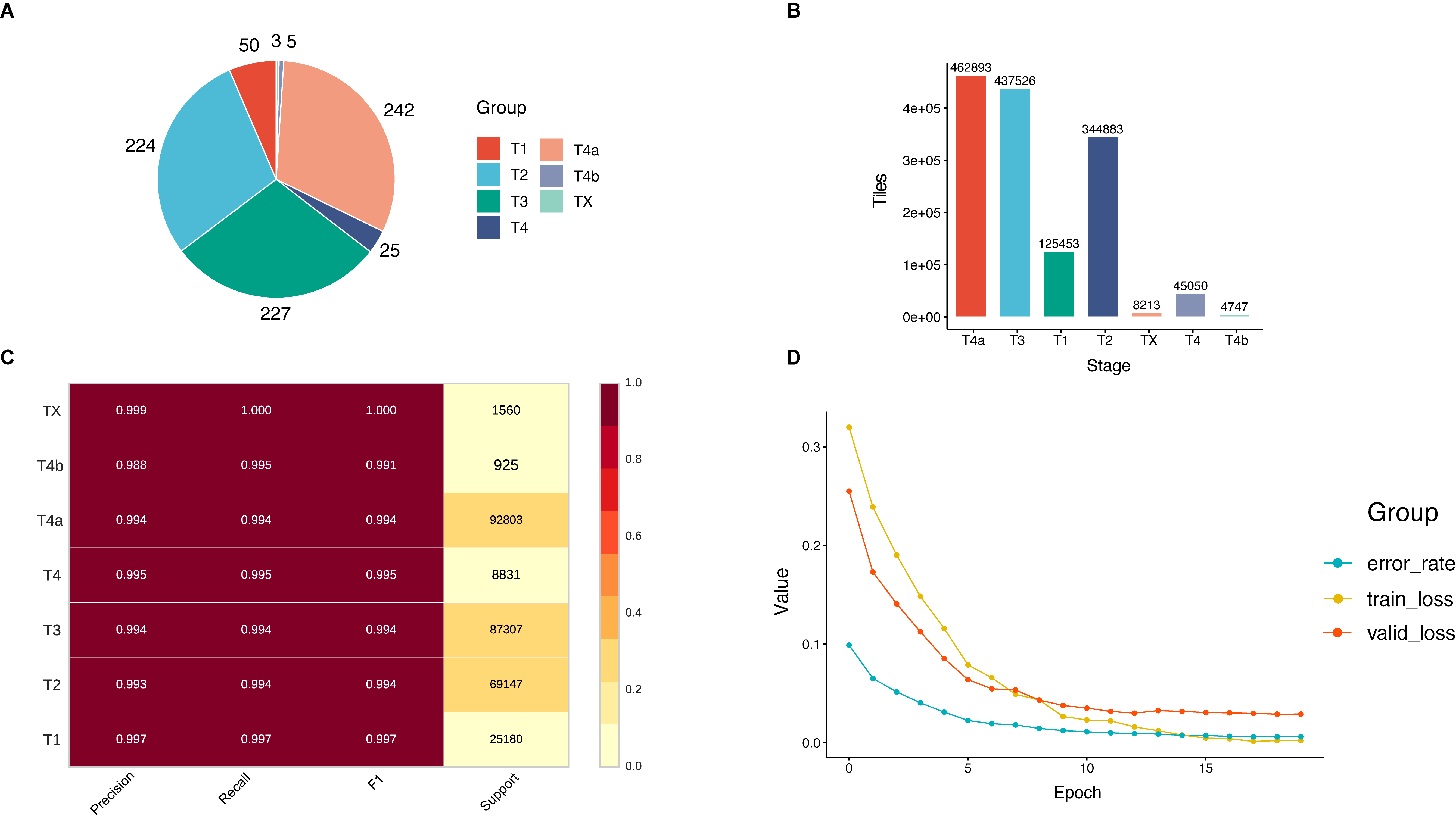

Supplement: Supplementary file 3 [file Image4.tif]

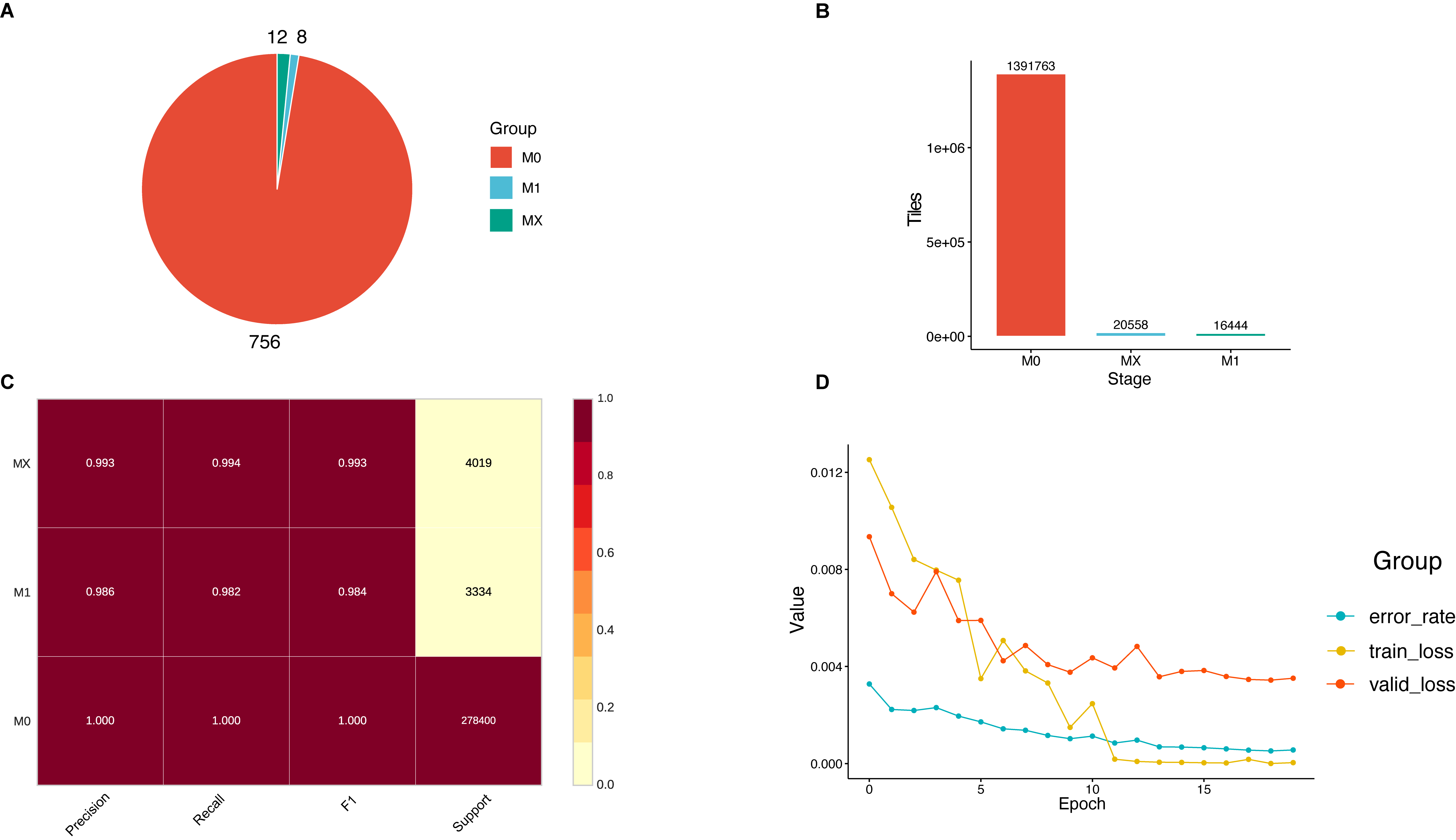

Supplement: Supplementary file 4 [file Image2.tif]

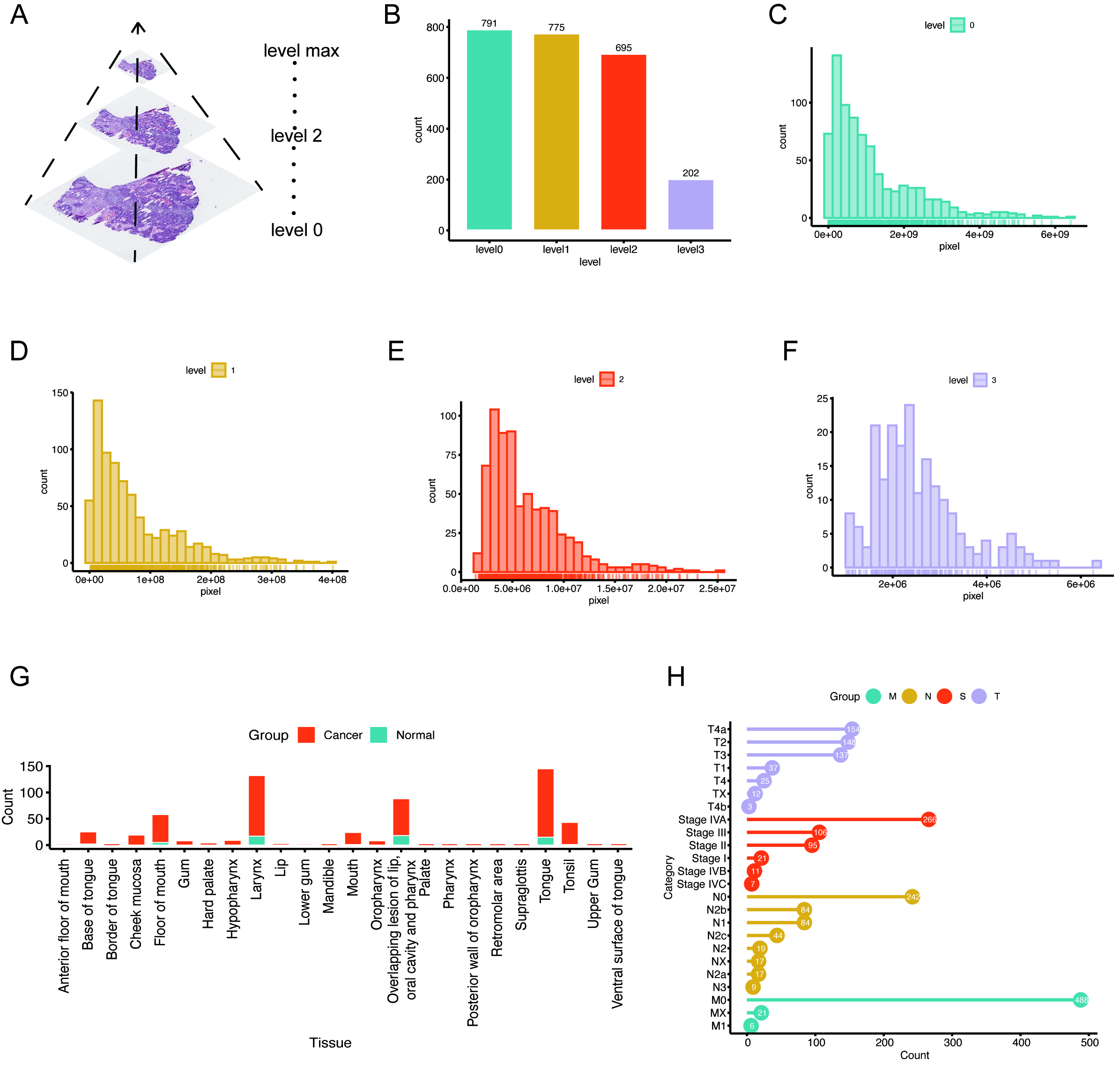

Supplement: Supplementary file 5 [file Image1.tif]

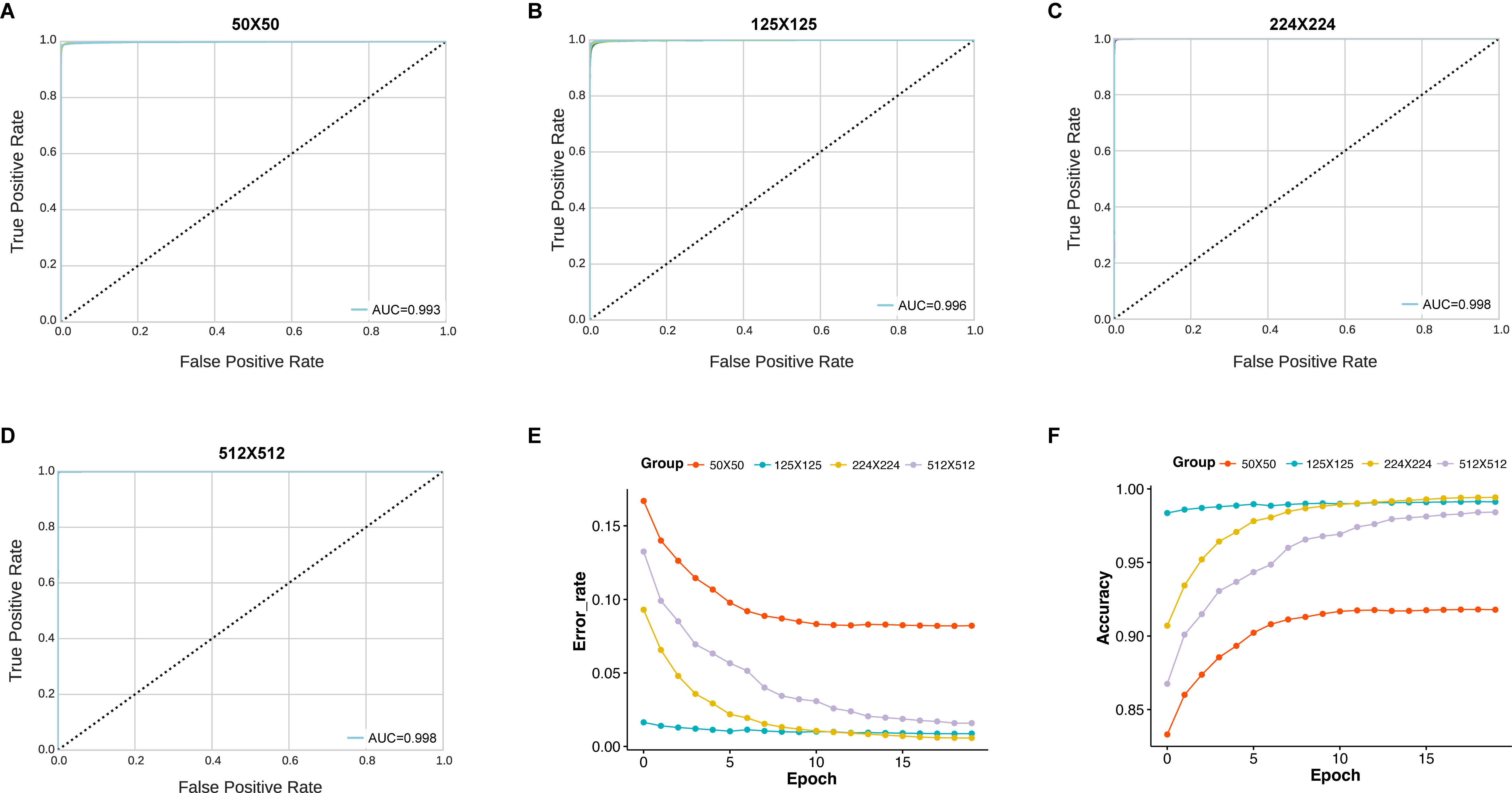

Supplement: Supplementary file 7 [file Image5.tif]
